# Event-Dataset: Temporal information retrieval and text classification dataset

**DOI:** 10.1016/j.dib.2019.104048

**Published:** 2019-05-23

**Authors:** Shafiq Ur Rehman Khan, Muhammad Arshad Islam

**Affiliations:** Capital University of Science and Technology, Islamabad, Pakistan

**Keywords:** Information retrieval, Temporal, Text classification, Focus time assessment

## Abstract

Recently, Temporal Information Retrieval (TIR) has grabbed the major attention of the information retrieval community. TIR exploits the temporal dynamics in the information retrieval process and harnesses both textual relevance and temporal relevance to fulfill the temporal information requirements of a user Ur Rehman Khan et al., 2018. The focus time of document is an important temporal aspect which is defined as the time to which the content of the document refers Jatowt et al., 2015; Jatowt et al., 2013; Morbidoni et al., 2018, Khan et al., 2018. To the best of our knowledge, there does not exist any standard benchmark data set (publicly available) that holds the potential to comprehensively evaluate the performance of focus time assessment strategies. Considering these aspects, we have produced the Event-dataset, which is comprised of 35 queries and set of news articles for each query. Such that, $C=\left\{Q_{s},\;D_{s}\right\},$ where C represents the dataset, $Q_{s}\;$is query set $Q_{s}=\left\{q_{1},\;q_{2},\;q_{3},\;\ldots \ldots .,\;q_{35}\right\}\;$and for each $q_{i}$ there is a set of news articles $q_{i}=\left\{d_{r},d_{nr}\right\}$. $d_{r},d_{nr}\;$are sets of relevant documents and non-relevant documents respectively. Each query in the dataset represents a popular event. To annotate these articles into relevant and non-relevant, we have employed a user-study based evaluation method wherein a group of postgraduate students manually annotate the articles into the aforementioned categories. We believe that the generation of such dataset can provide an opportunity for the information retrieval researchers to use it as a benchmark to evaluate focus time assessment methods specifically and information retrieval methods generically.

**Table:** Specifications table

Subject area	*Computer science*
More specific subject area	*Information retrieval, temporal information retrieval, text classification*
Type of data	*Text, spreadsheets*
How data was acquired	*Raw data were acquired using a web spider*
Data format	*Processed text files, spreadsheets*
Experimental factors	*Standard text preprocessing methods are applied.*
Experimental features	*Annotated by postgraduate students*
Data source location	*Islamabad, Pakistan (Latitude & Longitude* 33.6844° N, 73.0479° E)
Data accessibility	*Data is with this article*
Related research article	*S. U. R. Khan, M. A. Islam, M. Aleem, M. A. Iqbal, and U. Ahmed, “Section-Based Focus Time Estimation of News Articles,” IEEE Access, vol. 6, pp. 75,452–75460, 2018.*

**Table:** 

**Value of the data** Event-Dataset presents relevant news articles related to 35 popular events from the past and future and it has the following applications: • It can be used for information retrieval tasks. • It can be used for temporal information retrieval tasks. • It can be used to evaluate the methods of estimating the focus time of documents [5]. • It can be used for text classification purpose.

## 1 Data

**Fig. 1:**
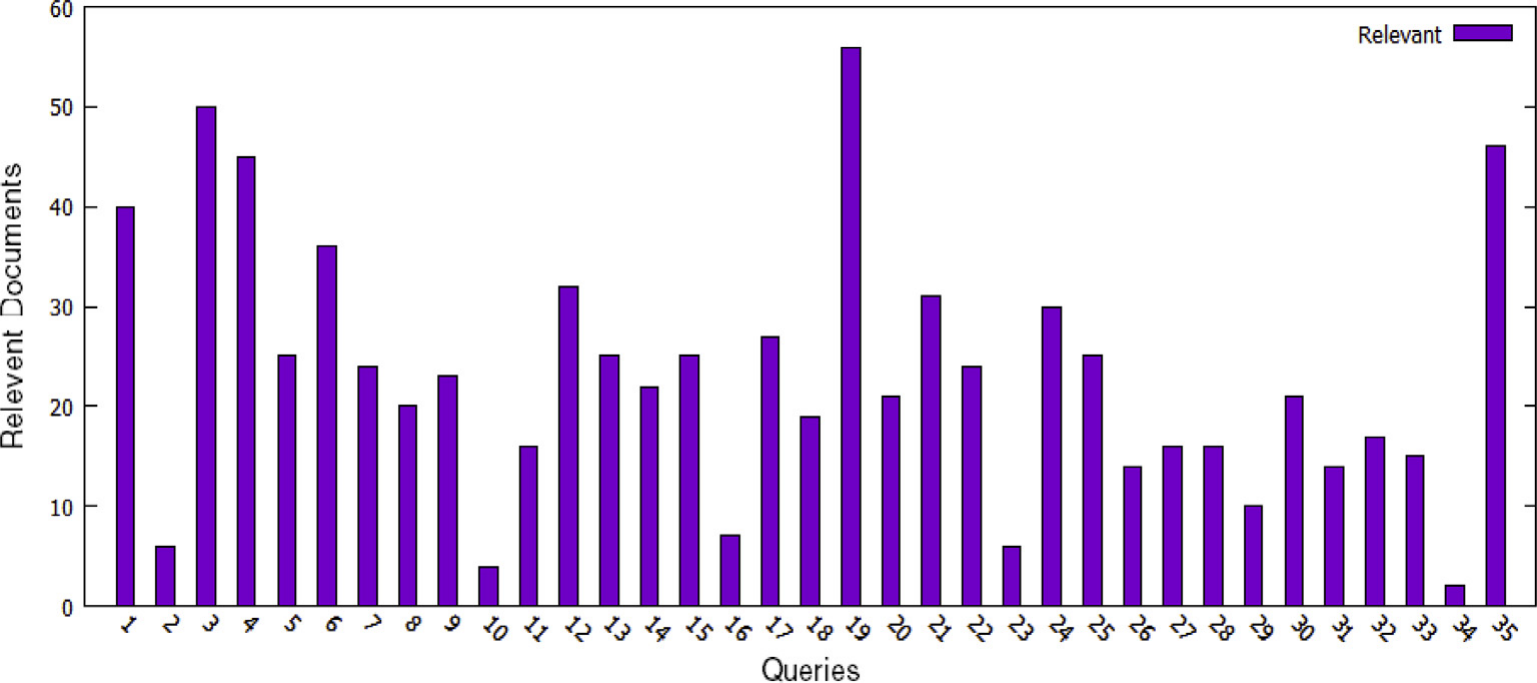
Distribution of relevant documents over individual events.

**Fig. 2:**
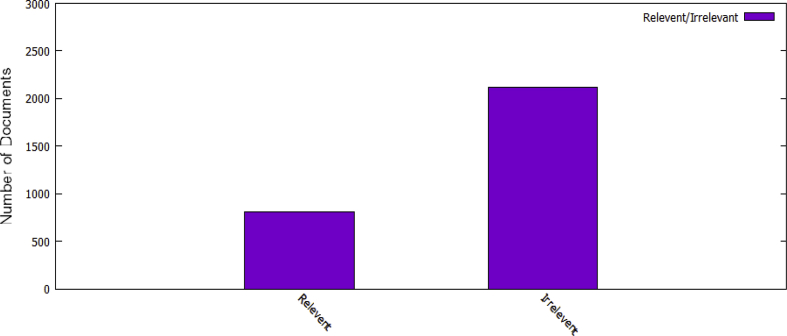
Total relevant and irrelevant documents in the annotated Events-dataset.

The Event-Dataset contains relevant and non-relevant news articles for 35 popular events (Table 1). The news articles are annotated by human annotators into relevant and irrelevant classes (Fig. 1, Fig. 2), providing rational for their judgment (Table 2). The Event-Dataset folder contains 35 subfolders and one spreadsheet file (queries.xlsx). The queries.xlsx file contains the set of 35 queries and the corresponding year and description. Each subfolder contains text files and an MS Excel spreadsheet. The text files are original news articles scrap from different news sources using Google News search API. The spreadsheet contains the user relevancy judgment where 0 mean not relevant and 1 means relevant.

**Table 1:** The description of events that are considered in the development of this dataset with the corresponding year.

Event ID	Event	Year	Description
1	Ambassador Steven Death	2012	U.S. Ambassador to Libya J. Christopher Stevens was among four Americans killed in an attack by Muslim protesters on the U.S. consulate compound in Benghazi the previous evening, the U.S. government confirmed Wednesday, 12 Sep 2012
2	Athens wildfire	2009	Fire erupts near Makri Village northeast of Athens on 24 August 2009.
3	Baltimore riots	2015	Freddie Gray, 27, of Baltimore, was arrested on April 12 and died on Sunday from a spinal injury after slipping into a coma. His death has sparked outrage and protests in the largely black Maryland city of about 625,000 people.
4	Benazir Assassination	2007	Pakistan's former Prime Minister Benazir Bhutto was assassinated at a large gathering of her supporters where a suicide bomber also killed at least 14 on 27 December 2007.
5	Pope Benedict XVI	2005	With unusual speed and a little surprise, Cardinal Joseph Ratzinger of Germany became Pope Benedict XVI on Tuesday (2005) a 78-year-old transitional leader who promises to enforce strictly conservative policies for the world's Roman Catholics."
6	BP Oil Spill	2010	An explosion rocked an offshore oil drilling platform, sending a column of fire into the sky and touching off a frantic search at sea Wednesday for 11 missing workers. Most of the 126 workers on the rig Deepwater Horizon escaped safely after the explosion about 10 p.m. Tuesday (03 August 2010)
7	Cricket World cup	2019	Cricket World cup will be held in 2019 in England.
8	David Cameron Resignation	2016	The former prime minister, 49, stepped down as a leader in June 2016 shortly after 52% of Britons ignored his pleas and voted to leave the European Union
9	Kashmir Earthquake	2005	Kashmir Earthquake in 2005 was the biggest tragedy in its history of Pakistan, leaving the devastated nation reaching out for help from around the world.
10	Fidel Castro Retirement	2008	Fidel Castro stepped down (Feb 2008) as the president of Cuba after a long illness. The resignation ends one of the longest tenures as one of the most all-powerful communist heads of state in the world.
11	FIFA Football World Cup	2022	Qatar will host the World Cup finals for the first time after FIFA awarded them the rights to the 2022 tournament in Zurich.
12	Fukushima Disaster	2011	A powerful explosion has hit a nuclear power station in north-eastern Japan which was badly damaged in Friday's (12 March 2012) devastating earthquake and tsunami.
13	Haiti Earthquake	2010	The disaster caused by the January 12, 2010 earthquake in Haiti is the worst in modern history, according to a new report by the Inter-American Development Bank (IDB). Up to 250,000 people have been killed, and up to $14 billion in damage has been caused by the quake, which rated a 7.0 on the Richter scale.
14	Hurricane Katrina	2005	Hurricane Katrina, one of the strongest storms ever to threaten the United States packed with 165-mph winds and forced the evacuation of hundreds of thousands of residents of New Orleans and the region.
15	Hurricane Sandy	2012	Hurricane Sandy, one of the largest and fiercest storms to menace the East Coast in years, slammed into New Jersey on Monday evening with torrential rains, howling winds, and widespread flooding 30 October 2012
16	Independence of South Sudan	2011	On July 9, African and international leaders gathered in Juba, the capital of South Sudan, to welcome the newest nation on earth. The former southern provinces of Sudan, South Sudan became the 54th nation of the African Union and the 193rd member of the United Nations.
17	London Bombing	2005	07 July 2005 a series of bomb attacks on London's transport network has killed more than 30 people and injured about 700 others. Three explosions on the Underground left 35 dead and two died in a blast on a double-decker bus.
18	Madrid Terrorist Attacks	2004	11 March 2004, powerful explosions have torn through three Madrid train stations during the morning rush hour with latest reports speaking of 173 people killed. Near-simultaneous blasts hit Atocha station in the center of the Spanish capital and two smaller stations.
19	MH370 Disappearance	2014	Almost 240 people are missing after a Malaysian Airlines flight en route from Kuala Lumpur to Beijing vanished from radar screens in the March 2014.
20	Michael Jackson Death	2009	The tragedy of Michael Jackson's death at age 50, reportedly from cardiac arrest, pales in comparison to the tragedy of his life on 25 June 2009
21	Mike Tyson “The Bite Fight”	1997	On June 28, 1997, Mike Tyson bites Evander Holyfield ear in the third round of their heavyweight rematch. The attack led to his disqualification from the match and suspension from boxing and was the strangest chapter yet in the champions roller-coaster career.
22	Moscow Terror Attack	2010	At least 38 people were killed and more than 60 injured in two suicide bomb attacks on the Moscow Metro during the morning rush hour.
23	Cyclone Nargis	2008	Cyclone Nargis made landfall in the Irrawaddy delta region, some 250 km southwest of Yangon around 4:00 p.m. on 2nd May 2008
24	Pakistan Flood	2010	The worst flood in the history of Pakistan in August 2010.
25	Pervaiz Musharraf resignation	2008	The resignation of Pakistan's Pervez Musharraf has been accepted with immediate effect by national lawmakers in August 2008
26	Prince Charles Wedding	2005	British heir to the throne Prince Charles finally marries long-time lover Camilla Parker Bowles in April 2005
27	Prince William Wedding	2011	2011Kate Middleton marries Prince William in April 2011.
28	Rayan Dunn Death	2011	“Jackass” star Ryan Dunn and a passenger in his car died of “blunt and thermal trauma” when the 2007 Porsche 911 GT3 crashed and caught fire on a Pennsylvania highway in June 2011
29	Tunisia Revolutions	2011	Tunisia revolution start in January 2011
30	Robbin Williams Death	2014	US actor and comedian Robin Williams has been found dead, aged 63, in an apparent suicide in August 2014.
31	Saddam Hussein Execution	2006	The former Iraqi leader Saddam Hussein has been hanged in northern Baghdad for crimes against humanity in December 2006.
32	Sochi Olympics	2014	President Vladimir Putin on Friday 8th February 2014 opened the Winter Olympics Games in Sochi that are inextricably linked with his name, after a stunning ceremony where Russia sought to convince the world it is a worthy host.
33	Steve Jobs Death	2011	Steve Jobs, co-founder, and former chief executive of US technology giant Apple, has died at the age of 56 in October 2011.
34	Switzerland Joined the UN	2002	In 2002, Switzerland joins the United Nations.
35	Volkswagen Scandal	2015	Volkswagen says 11 million vehicles worldwide are involved in the scandal that has erupted over its rigging of US car emissions tests. It said it was setting aside 6.5bn (4.7bn) to cover costs of the scandal.

**Table 2:** Example of annotation by 2 annotators.

Event	Description			
Baltimore riots (2015)	Freddie Gray, 27, of Baltimore, was arrested on April 12 and died on Sunday from a spinal injury after slipping into a coma. His death has sparked outrage and protests in the largely black Maryland city of about 625,000 people.			
Annotator	Document 1		Document 2	
	Relevancy	Rational	Relevancy	Rational
Annotator 1	1	The incident is not discussed in detail. It is about the protests after Freddy's death and the trial of the police officer. So it seems relevant.	0	This news is about the Protest against Trump Policies.
Annotator 2	1	Information about the judge remarks in Freddy's death case.	0	The article discusses some protest again president Donald Trump. Completely irrelevant news article.

**Table 3:** Event-dataset description.

Dataset	Events dataset
Source	Google News
Purpose	Document focus time estimation
Type	News (text)
Total Events	35
Future events (after 2018)	2
Past events (before 2017)	33
Total queries (4/event)	140
Annotators (for each event documents)	2
Year range (events)	1997–2022
Year range (creation time)	2000–2017
Total relevant	810
Total irrelevant	2116
Total documents	2926
Data extraction year	2017

For each event, news articles are collected using Google News search. A web scraper is developed to extract the documents, where the scraper searches the query in Google news search. The queries are comprised of events discussed in the following section. The extracted documents are then annotated by postgraduate students. In the annotation process, each article is observed by two participants for the relevancy, i.e., either relevant to the event or not. The annotators are then asked to justify their judgment. This dataset is developed with an intention to determine focus time of news articles [1], [2], [3], [4]. The Event-dataset can also be used for general information retrieval and text classification tasks (Table 4). The dataset contains 35 temporal queries and a set of relevant and non-relevant news documents.

**Table 4:** Categorization of 35 events into 4 classes.

Environmental	Politics	Violence	Entertainment and Sports
Athens wildfire, 2009-Q2	David Cameron Resignation, 2016	London Bombing, 2005	Michael Jackson Death, 2009
BP Oil Spill, 2010	Fidel Castro Retirement, 2008	Madrid Terrorist Attacks, 2004	Prince Charles Wedding, 2005
Kashmir Earthquake, 2005	South Sudan Independence, 2011	MH370 Disappearance, 2014	Prince William Wedding, 2011
Fukushima Disaster, 2011	Pervaiz Musharraf resignation, 2008	Moscow terror attack, 2010	Rayan Dunn Death, 2011
Haiti Earthquake, 2010	Tunisia Revolutions, 2011	Benazir Assassination, 2007-Q4	Robbin Williams Death, 2014
Hurricane Sandy, 2012	Switzerland Joined UN, 2002	Baltimore riots, 2015-Q3	Steve Jobs Death, 2011
Hurricane Katrina, 2005	Pope Benedict XVI, 2005-Q5	Saddam Hussein Execution, 2006	Cricket World cup, 2019
Cyclone Nargis, 2005		Ambassador Steven Death, 2012	FIFA Football World Cup, 2022
Pakistan Flood, 2010			Mike Tyson “The Bite Fight”,1997
Volkswagen Scandal, 2015			Sochi Olympics, 2014

## 2 Experimental design, materials and methods

35 popular events from the past and future are selected that occurred during the years of 1997–2022. The events are well reported all over the world and selected randomly. www.brainyhistory.com website is used to verify the events. This website maintains a list of popular events from year 1–2015 AC. A couple of future events are related to sports events, such as Football and Cricket world cup. Google trend tool (www.trends.google.com) can also be used to verify the popularity of events from the year 2008 to the current year. The events, the corresponding year and a brief description is presented in Table 1.

**Queries:** In order to retrieve the most relevant documents, we use explicit temporal queries $Q_{t}$, $\;Qt\;=\;\left\{Q_{text},\;\;Q_{time}\right\}$ comprises of two parts: a textual part $Q_{text}$ and temporal part $Q_{time}$, where $Q_{text}\;=\;\left\{W_{1},\;W_{2},\;W_{3}\ldots ..W_{n}\right\}$ and $Q_{t}=\left\{Year_{e}\right\}$. The textual part $Q_{text}$ comprises of query terms (i.e., event name) and the temporal part$Q_{time}$ is the year when the event occurred. Such queries are normally referred to as explicit temporal queries. Explicit temporal queries capture the real world meaning of time [6]. For instance, to collect relevant news documents pertaining to an event of Prince Charles wedding, the query is “Prince Charles Wedding 2005". Multiple related queries are used to extract the event related news articles. For example, for BP Oil Spill event the queries were " BP oil spill 2010″, “Deepwater Horizon oil spill 2010″, “BP oil disaster 2010″, “Gulf of Mexico oil spill 2010″ and “Macondo blowout 2010".

**Platform and Process:** Google News API is used to extract the document collection using a spider. The queries for events are searched in Google News using API. The crawler is designed in such a way that the top 100 news are extracted against the individual query. As a single event has multiple related queries, the probability of retrieving duplicate documents is high. To address this problem, the crawler discards all the duplicate document and only downloads unique documents. The crawler searches for three types of information. i.e. the title of a news story, creation date and text content of the original story. In some pages, if the creation time is not available, the publication time or updating time is used as creation time. The top k news articles (k = 100) ranked by the Google news search are extracted against each event. After removing the duplicate documents, a total of 2926 news documents against 35 queries is collected for the dataset.

**Preprocessing:** Standard preprocessing methods are used to clean the data. These methods include: removing unwanted text, conversion to lower case, removing duplicate documents, removing documents have wrong creation time and removing hyperlinks or images descriptions.

**Annotation:** A gold standard is developed by relying on human judgments in identifying the actual focus time from news documents. Total of 2926 news documents (related to 35 events) is distributed among 70 post-graduate students. Each participant is assigned the news documents related to a specific event (query) and asked to label each document as relevant or non-relevant according to the given event. Thus, for each event, the extracted news documents are labeled by 2 participants. The relevance of a document to the query obviously ensures that the document relates to a corresponding event (i.e., event presented in the query). If annotators discovered that the document content is dominated by the main event [7], they marked it as relevant, otherwise non-relevant. The event relevant documents in the dataset follow the notation “news-peg" [8], which is defined as an event which prompted the author to the article. News-peg serves as a measure of noteworthiness, estimating the role of event importance in prompting the author to write an article.

The participants were asked to provide the rational for each judgment. The annotator rational is in the form of the short excerpt (2 or 3 sentences), explaining why the annotator think a document is relevant or irrelevant. This method of the annotation is proved to be efficient in Information Retrieval (IR) tasks and incurs no additional time as the annotator might be already doing so implicitly [9], [10]. Table 2 shows an example of rationales for two documents. Finally, we consider those documents to be relevant where both the participants agreed with respect to the temporal annotation. Whereas, documents are irrelevant if both annotators mark it as irrelevant or have conflicting remarks. For 771 (26.34%) documents the annotators have conflicting remarks whereas, for 2155 (73.65%) documents the annotators have the same remarks. Out of these 2155 documents, 810 (27.68%) documents are marked as relevant whereas, 1345 (45.96%) are marked as irrelevant. Table 3 shows the Event-Dataset description. The relevant documents against each individual event in the dataset are presented in Fig. 1. $Q_{1\;},\;Q_{2},\ldots \ldots ..,\;Q_{35}$ on X-axis shows the events in alphabetic order as described in Table 1. Fig. 2 presents the statistics of relevant and non-relevant documents in the dataset.

The Event-dataset can also be used for classification tasks. The selected events can be categorized into high-level categories. Table 4 shows 35 events into 4 high-level classes including, environmental, political, violence and entertainment and sports. Event detection and topic modeling techniques can be evaluated using Event-dataset. For such purpose, the relevant documents for each event can be used to test topic modeling and event detection methods.
